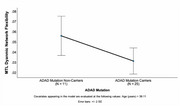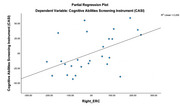# Medial Temporal Lobe Dynamic Network Flexibility is Reduced Among Autosomal Dominant Alzheimer's Disease Mutation Carriers

**DOI:** 10.1002/alz70856_106881

**Published:** 2026-01-13

**Authors:** Wiktoria Piaszczynska, Miray Budak, Bryan Rowe, Soodeh Moallemian, Bernadette A. Fausto, John M Ringman, Mark A. Gluck

**Affiliations:** ^1^ Center for Molecular & Behavioral Neuroscience, Rutgers University–Newark, Newark, NJ, USA; ^2^ Rutgers University–Newark, Newark, NJ, USA; ^3^ Keck School of Medicine, University of Southern California, Los Angeles, CA, USA

## Abstract

**Background:**

Autosomal Dominant Alzheimer's disease (ADAD) due to mutations in *PSEN1, APP*, and *PSEN2* offers a unique framework for elucidating the neuropathological mechanisms underlying early disease stages. Given the critical role of the MTL in memory and cognitive processing, the ADAD mutations have yet to be thoroughly investigated regarding its impact on medial temporal lobe (MTL) dynamics and subfield‐specific integrity in FAD. This study focuses on identifying biomarkers for early disease progression by examining ADAD mutations on MTL network flexibility, subfield volumetric integrity, and associated cognitive outcomes among autosomal dominant AD.

**Method:**

The cohort comprised 36 participants (28 women) carrying ADAD mutations (*Mean_age_=39.11, SD_age_=12.16*) who underwent genotyping, cognitive assessment using the Cognitive Abilities Screening Instrument (CASI), and brain neuroimaging with a Siemens Magnetom 3T Trio scanner. ANCOVA and Linear Regression analysis were applied using age as a covariate.

**Result:**

ADAD mutation carriers exhibited significantly reduced MTL network flexibility (*F*_(1)_= 4.551, 𝜂_p_^2^= .121, *p* = .040), indicating altered dynamic connectivity, despite no observed volumetric differences in structural analyses (*p* > .05). Notably, volumetric measures of the right entorhinal cortex were positively correlated with CASI scores (*b* = .492, t = 2.837, *p* = .009).

**Conclusion:**

Among ADAD mutation carriers, reduced MTL dynamic network flexibility suggesting early functional changes despite no significant differences in subfield volumes. Additionally, larger right entorhinal cortex volume was associated with better cognitive performance, highlighting its role in preserving cognitive function. These findings suggest that dynamic functional network alterations within the MTL region may serve as early indicators of disease progression in ADAD, even before structural atrophy becomes apparent. This study also underscores the need for integrating network‐based and subfield‐specific analyses while considering ADAD mutation status to better understand disease mechanisms.